# Fluctuating hypertension possibly leading to severe cardiomyopathy in SDHB-associated hereditary paraganglioma: from heart transplant candidacy to long-term lanreotide management: a case report

**DOI:** 10.3389/fcvm.2026.1861073

**Published:** 2026-07-30

**Authors:** Emily Pack, George Sokos, Christopher Bianco, Brittany Carey, Adnan Haider, Alan Thomay, Xiaofei Wang, Sonikpreet Aulakh

**Affiliations:** 1 West Virginia University School of Medicine, Morgantown, WV, United States; 2 Department of Cardiology, West Virginia University, Morgantown, WV, United States; 3 Department of Endocrinology, West Virginia University, Morgantown, WV, United States; 4 Department of Surgical Oncology, West Virginia University, Morgantown, WV, United States; 5 Department of Radiology, West Virginia University, Morgantown, WV, United States; 6 Department of Medical Oncology, West Virginia University, Morgantown, WV, United States

**Keywords:** case report, catecholamine cardiomyopathy, heart failure, lanreotide, neuroendocrine tumor, paraganglioma, SDHB

## Abstract

**Introduction:**

Paragangliomas are rare catecholamine-secreting neuroendocrine tumors that can cause significant cardiovascular complications, including catecholamine-induced cardiomyopathy. While surgical resection is the standard of care, some patients are not candidates for surgery or heart transplantation due to tumor biology, genetic risk, or patient preference.

**Case summary:**

A 68-year-old man with longstanding hypertension, hereditary paraganglioma–pheochromocytoma syndrome (SDHB mutation), and advanced nonischemic cardiomyopathy was evaluated for heart transplantation. Imaging revealed a highly vascular retroperitoneal paraganglioma, and biochemical testing demonstrated markedly elevated chromogranin A with mildly increased plasma free normetanephrine. Biopsy confirmed paraganglioma. Due to transplant ineligibility related to malignancy risk and the patient’s decision to decline surgical resection, medical therapy with lanreotide was initiated. Following treatment, the patient demonstrated biochemical improvement with declining chromogranin A levels, stabilization of blood pressure, improvement in B-type natriuretic peptide, modest recovery of left ventricular ejection fraction, and reduced metabolic activity on serial somatostatin receptor PET/CT imaging, with stable tumor size. Functional status remained excellent without further heart failure hospitalizations.

**Conclusion:**

This case highlights lanreotide as a potential non-surgical therapeutic option for paraganglioma-associated cardiomyopathy in select high-risk patients. Systemic tumor control may contribute to cardiovascular stabilization when surgical or transplant options are not feasible.

## 1 Introduction

Paragangliomas are rare catecholamine-secreting neuroendocrine tumors arising from sympathetic or parasympathetic extra-adrenal autonomic paraganglia, with an estimated incidence of 500–1,000 cases per year in the United States (1). Sympathetic paragangliomas most commonly occur in the abdomen, particularly near the junction of the inferior vena cava and left renal vein or at the organ of Zuckerkandl, but may arise anywhere along the sympathetic chain, including the thorax and pelvis (1). Parasympathetic paragangliomas most commonly arise from the carotid body, but may also occur in the jugular foramen, the middle ear and the vagus nerve (1). Paragangliomas have a median age at diagnosis of 47 years, with head and neck tumors presenting later and abdominal tumors often presenting in patients in their thirties. Patients with hereditary paraganglioma syndromes tend to present at a younger age than those with sporadic disease (1). Hereditary paragangliomas occur equally in both men and women while sporadic paragangliomas are more common in females than men (1).

Sympathetic paragangliomas most commonly secrete norepinephrine, activating the sympathetic nervous system and causing episodic hypertension, palpitations, and profuse sweating, though the classic triad occurs in only about 40% of patients (1). While most paragangliomas are sporadic, up to 35% are associated with hereditary mutations, particularly in genes encoding subunits of succinate dehydrogenase (SDH) (1).

Excess catecholamine secretion can lead to significant cardiovascular complications. The existing literature is limited primarily to case reports and small series describing cardiac complications in this setting. Reported manifestations include myocardial infarction, acute decompensated heart failure, and stress (takotsubo-like) cardiomyopathy (2–4). Early recognition is critical, as cardiovascular injury may be reversible with appropriate management.

We present a case of a 68-year-old man with non-ischemic cardiomyopathy with hereditary pheochromocytoma-paraganglioma syndrome managed exclusively with lanreotide, highlighting the challenges and potential efficacy of non-surgical systemic therapy in this rare and high-risk population.

## 2 Case

A 68-year-old male with an Eastern Cooperative Oncology Group (ECOG) performance status of 0 had a medical history notable for longstanding hypertension, nonischemic cardiomyopathy with heart failure with reduced ejection fraction (HFrEF), bicuspid aortic valve disease with mixed stenosis and regurgitation, and ventricular arrhythmias managed with cardiac resynchronization therapy-defibrilator. He was retired and previously physically active, engaging in regular cycling. His father died from heart failure in his early 30s, with no known history of neuroendocrine tumors.

The patient was initially diagnosed with systolic heart failure approximately 16 years prior, in the context of fluctuating hypertension treated with antihypertensives. He remained clinically stable for many years but developed progressive exertional intolerance, volume overload, and recurrent heart failure hospitalizations beginning in 2019. Patient’s blood pressure ranged from systolic of 130s−90s and diastolic of 80s−60s. Heart rate for patient has remained relatively stable since earliest report in 2018 with average staying around 77. However, patient had previously reported feeling palpitations in 2020–2022. A 14-day holter monitor report in 2021 showed a PVC burden of 16% and in 2022 of 17.7%. By late 2023, his condition progressed to advanced HFrEF with declining exercise tolerance, increasing diuretic requirements, and a left ventricular ejection fraction (LVEF) of 10%–15%, prompting referral for advanced heart failure therapies and heart transplant evaluation.

**Figure 1:**
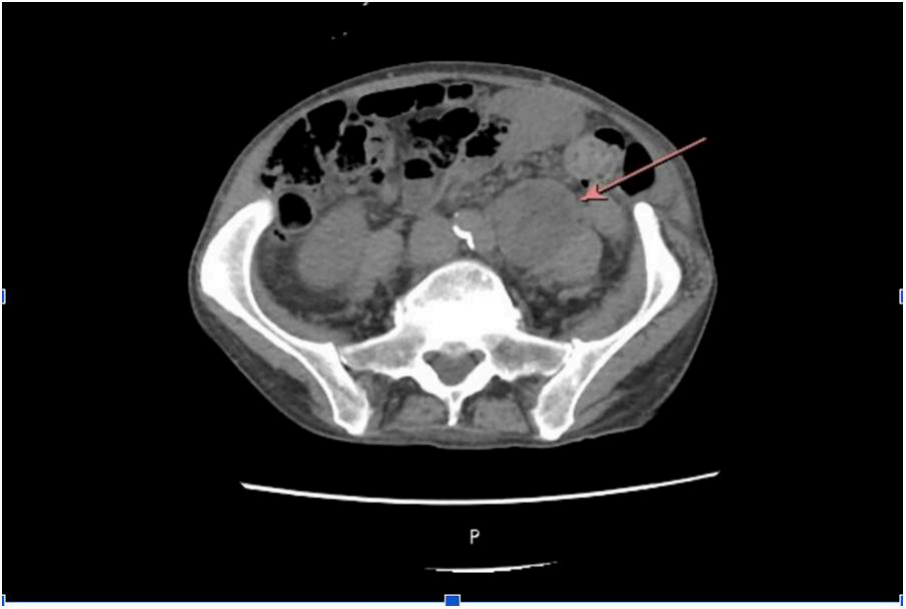
Initial diagnostic CT (November 2023). **Legend:** Axial contrast-enhanced CT of the abdomen and pelvis obtained at initial presentation (November 2023), demonstrating a well-circumscribed soft tissue mass (arrow) that prompted further diagnostic workup.

As part of transplant evaluation, a computed tomography (CT) scan of the abdomen and pelvis with contrast revealed an incidental retroperitoneal mass measuring approximately 4.3 × 4.7 × 5.0 cm in the left pelvis/retroperitoneum, abutting the aortic bifurcation, left common iliac artery, and adjacent vascular structures (Figure 1). The lesion appeared highly vascular, raising concern for malignancy and resulting in suspension of transplant candidacy pending oncologic evaluation.

In the winter of 2023, endoscopic ultrasound–guided biopsy of the mass demonstrated a neuroendocrine neoplasm consistent with paraganglioma. No mitoses were identified on tumor cells and were immunoreactive for synaptophysin, chromogranin, INSM1, inhibin, and GATA3, with rare S-100–positive sustentacular cells. Tumor cells were negative for CK AE1/AE3, CK7, CK20, PAX-8, CD45, MART-1, WT-1, calretinin, DOG1, and c-KIT. CARIS Tumor Seek analysis revealed a pathogenic SDHB variant, consistent with Hereditary Paraganglioma-Pheochromocytoma Syndrome. Natera germline testing confirmed the pathogenic SDHB variant.

In spring 2024, biochemical testing demonstrated mildly elevated plasma free normetanephrines (202 pg/mL, normal value <148 pg/mL) in the supine position and a normal 24-hour urine fractionated normetanephrines (439 µg/24 h). Plasma dopamine levels were less than 20 pg/mL (<27 pg/mL). Urine dopamine level 131 mcg/24 h (65–400 mcg/24 h). Biochemically the mild elevation of plasma normetanephrine suggested a minimally functional or clinically silent paraganglioma. Chromogranin A was markedly elevated at 1,595 and 1,588 ng/mL (reference <311 ng/mL). Patient underwent a Cu-64 DOTATATE positron emission tomography-computed tomography (PET-CT) scan without contrast, which showed a hypermetabolic left pelvic mass measuring 5.0 × 4.7 × 5.2 cm abutting the aorta, left common iliac artery and left psoas muscle. Prazosin was titrated to 2 mg twice daily to provide alpha blockade.

A multidisciplinary team including heart failure cardiology, oncology, endocrinology, and surgery determined the patient was a surgical candidate; however, he elected not to pursue tumor resection. Multidisciplinary evaluation also concluded that heart transplantation could not be pursued due to the presence of an SDHB-associated neuroendocrine tumor. The patient instead decided to pursue medical management with initiation of lanreotide injections every four weeks, initiated in January 2025.

**Figure 2:**
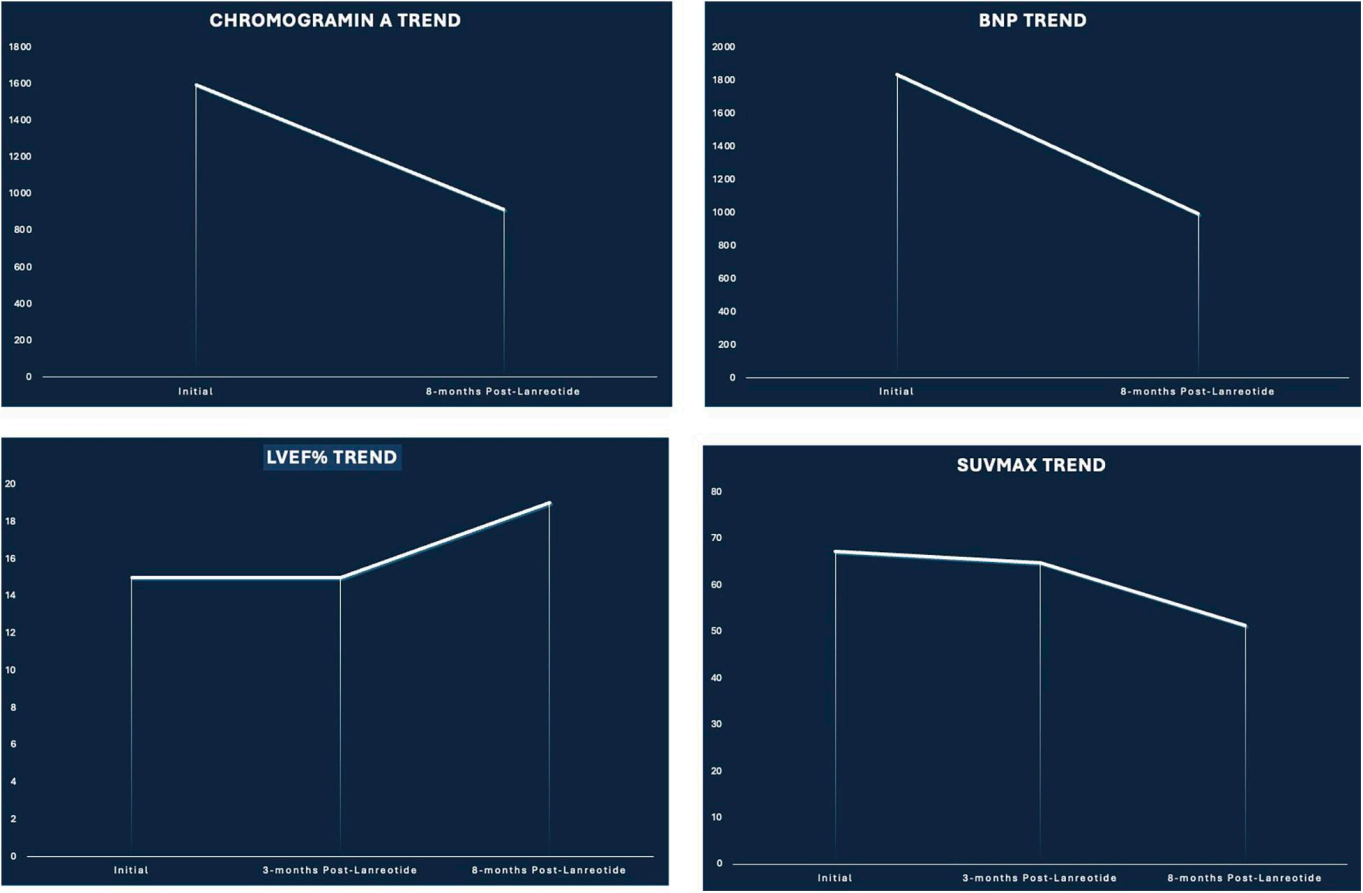
Biochemical, cardiac, and imaging response to lanreotide therapy. Legend: Trends in tumor markers, cardiac biomarkers, cardiac function, and metabolic imaging activity following initiation of lanreotide therapy. Serum chromogranin A declined from initial presentation to 8 months post-lanreotide. B-type natriuretic peptide (BNP) declined over the same interval. Left ventricular ejection fraction (LVEF%) improved initial presentation through 3 and 8 months post-lanreotide. Maximum standardized uptake value (SUVmax) on ⁶⁸Ga-DOTATATE PET/decreased at 3 and 8 months post-lanreotide relative to baseline.

**Figure 3:**
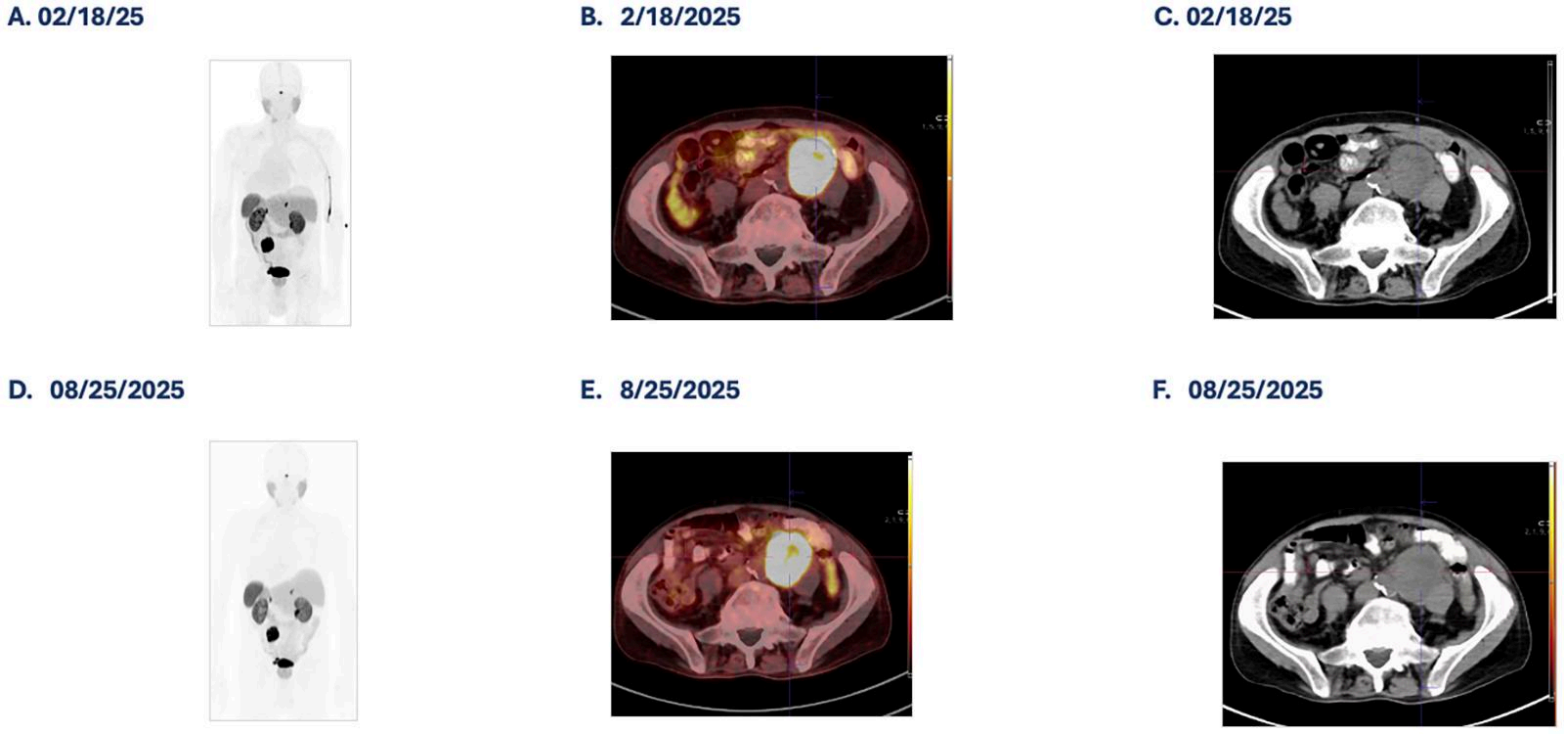
**(A)** whole-body ⁶⁸Ga-DOTATATE PET maximum-intensity projection image obtained prior to initiation of lanreotide therapy, demonstrating a somatostatin receptor-avid mass with expected physiologic tracer uptake in the kidneys, liver, spleen, and bladder. **(B)** Axial fused ⁶⁸Ga-DOTATATE PET/CT images at the level of the mass, obtained at baseline, demonstrating intense somatostatin receptor expression corresponding to the lesion identified on whole-body imaging. **(C)** Axial CT obtained at baseline, prior to lanreotide therapy (2/18/2025), shown for comparison. **(D) (**Whole-body ⁶⁸Ga-DOTATATE PET maximum-intensity projection image obtained 6 months post-lanreotide therapy, demonstrating a somatostatin receptor-avid mass with expected physiologic tracer uptake in the kidneys, liver, spleen, and bladder. **(E)** Axial fused ⁶⁸Ga-DOTATATE PET/CT images at the level of the mass, obtained six months post-lanreotide therapy demonstrating intense somatostatin receptor expression corresponding to the lesion identified on whole-body imaging. **(F)** Axial CT obtained six months post-lanreotide therapy (2/18/2025), shown for comparison.

Serial transthoracic echocardiograms demonstrated left ventricular systolic dysfunction prior to oncologic therapy, with LVEF declining from 20%–25% to 10%–15%, accompanied by left ventricular dilation and mixed aortic valve disease. Following initiation of lanreotide 120 mg subcutaneously every four weeks in January 2025, repeat echocardiography demonstrated stability in LVEF, with stable chamber size, valvular function, and right ventricular parameters. BNP levels improved from a peak of 1,837 pg/mL to 993 pg/mL and chromogranin A decreased to 915 ng/mL after eight months of treatment, reflecting a biochemical response to somatostatin analog therapy (Figure 2). Blood pressure stabilized with outpatient readings averaging 100s/60s mmHg, facilitating optimization of heart failure medications. Serial somatostatin receptor PET/CT imaging demonstrated stable PET avidity of the mass over time, with SUVmax around 60s after treatment initiation (Figure 3). A recent 14-day Holter monitor in 2025 showed a PVC burden of 2.8% a marked decrease since treatment.

The patient continues lanreotide and prazosin therapy with stable clinical status (ECOG 0), biochemical improvement, and no radiographic progression on serial somatostatin receptor PET/CT imaging (Table 1).

**Table 1:** Clinical timeline of disease progression, diagnosis, and management.

Timeframe	Clinical Events & Findings	Diagnostics	Interventions	Outcomes
∼2008 (16 years prior)	Initial diagnosis of systolic heart failure in setting of fluctuating hypertension	Echocardiogram (reduced EF, details not available)	Guideline-directed medical therapy (GDMT)	Clinically stable for several years
2019	Worsening exertional intolerance, volume overload, recurrent HF hospitalizations	Serial echocardiograms showing progressive LV dysfunction	Escalation of HF therapy	Gradual decline in functional status
Fall 2023	Advanced HFrEF; evaluation for heart transplantation	Echocardiogram: LVEF 10%–15%	Referral for transplant evaluation	Severe functional limitation
Late 2023	Incidental retroperitoneal mass discovered during transplant workup	CT abdomen/pelvis: 4.3 × 4.7 × 5.0 cm vascular mass	Transplant evaluation paused	Concern for malignancy
Winter 2023	Tissue diagnosis established	EUS-guided biopsy: paraganglioma; positive synaptophysin, chromogranin; SDHB mutation confirmed (CARIS + germline testing)	Multidisciplinary evaluation	Diagnosis of hereditary PPGL
Spring 2024	Biochemical and functional tumor assessment	Chromogranin A ∼1,595 ng/mL; mildly elevated plasma normetanephrine; Cu-64 DOTATATE PET/CT showing avid mass	Initiation of alpha-blockade (prazosin)	Partial BP control
Late 2024	Treatment decision-making	Multidisciplinary review	Patient declined surgery; deemed ineligible for transplant	Transition to medical management
January 2025	Initiation of systemic therapy	Baseline labs: elevated BNP and chromogranin A	Lanreotide 120 mg every 4 weeks	Start of biochemical and clinical response
∼8 months post-treatment (2025)	Clinical reassessment	↓ Chromogranin A (to 915 ng/mL); ↓ BNP (1,837 → 993 pg/mL); PET SUVmax decline (67.3 → 51.4); Echo LVEF stable around19%	Continued lanreotide + prazosin	Stabilized BP, improved EF, no HF hospitalizations
Ongoing (2025–present)	Stable disease and functional status	Serial PET/CT: stable tumor size, decreased avidity	Continued lanreotide therapy	ECOG 0, sustained clinical stability

## 3 Discussion

Pheochromocytoma and paraganglioma (PPGL)–associated cardiomyopathy is a rare but clinically significant manifestation of chronic catecholamine excess. Cardiomyopathy occurs in approximately 10%–15% of PPGL cases and may present as dilated cardiomyopathy, hypertrophic remodeling, or stress-induced (takotsubo-like) cardiomyopathy (5, 6). The pathophysiology involves prolonged catecholamine-mediated myocardial injury, including coronary microvascular dysfunction, oxidative stress, intracellular calcium overload, and direct myocyte necrosis, which may ultimately progress to irreversible ventricular remodeling and systolic dysfunction (5, 7).

In the present case, the patient’s long-standing labile hypertension and progressive nonischemic cardiomyopathy possibly preceded the diagnosis of paraganglioma by more than a decade. Such diagnostic delay is common, as the classic triad of headache, palpitations, and diaphoresis is present in fewer than half of patients, and cardiovascular manifestations may dominate the clinical presentation (8). Chronic catecholamine exposure from an occult tumor may therefore result in insidious and underrecognized myocardial injury.

While surgical resection remains the primary treatment for paragangliomas, a subset of tumors are locally unresectable due to vascular or neural involvement, metastatic spread, significant comorbidities, or patient preference. In such cases, management focuses on symptom control, biochemical suppression, and tumor stabilization through systemic and locoregional therapies within a multidisciplinary framework. Alpha-adrenergic blockade remains essential for symptom control in hormonally active tumors (4).

Plasma-free normetanephrines were only mildly elevated, despite the large size of the paraganglioma, and biopsy of this mass did not cause a hypertensive crisis, providing circumstantial evidence that this paraganglioma may have been low-secreting or only minimally functional. Biochemically silent paraganglioma is reported in some patients with SDH-B mutation, where tumors may lack tyrosine hydroxylase, the rate-limiting enzyme in catecholamine synthesis (4).

Radiation-based approaches may provide local control for unresectable disease. External beam radiation therapy or stereotactic body radiotherapy can be used for local tumor control and symptom palliation, particularly for skull base or paraspinal lesions (9). Among systemic options, somatostatin analogs (e.g., lanreotide or octreotide) are used in patients with somatostatin receptor (SSTR)–positive tumors for biochemical control and potential tumor stabilization, with emerging prospective data supporting their role in selected pheochromocytoma/paraganglioma patients (9). Although data remain limited because of the rarity of PPGL, increasing prospective evidence supports the use of somatostatin analogs. The phase II LAMPARA study has demonstrated encouraging disease control rates and acceptable safety with lanreotide in metastatic or unresectable PPGL (10). Lanreotide was well tolerated with 6.5% experiencing a grade 3 and less than 1% grade 4 adverse events (AEs) and no unexpected toxicities (10). Median PFS exceeded 2 years, with a median follow-up of 40 months (10). Patients treated with lanreotide or octreotide analogues have experienced disease stabilization or biochemical improvement, with durations of control ranging from several months to over 2–3 years in some reports (e.g., stable disease observed for up to 36 months or longer in individual cases of head and neck paragangliomas) (5, 11, 12). Peptide receptor radionuclide therapy (PRRT) with lutetium Lu-177 DOTATATE is an established option for patients with unresectable or metastatic SSTR-positive paragangliomas and has demonstrated meaningful disease control rates and symptomatic benefit (9, 13). PRRT (such as with 177Lu-DOTATATE) has demonstrated favorable outcomes in phase II trials with a median progression-free survival of 19.9 months and median overall survival of 51.7 months (13). Collectively, this data suggests that patients with SSTR-expressing tumors may derive benefit from therapeutic strategies targeting the somatostatin receptor pathway.

Targeted systemic therapies are also used, particularly in progressive disease (9). These include tyrosine kinase inhibitors (such as sunitinib, cabozantinib, or pazopanib) targeting angiogenic pathwaysIn tumors associated with hypoxia pathway alterations (e.g., SDHx- or EPAS1-related disease), HIF-2*α* inhibitors can be used for metastatic PPGL (9). Conventional cytotoxic chemotherapy (such as temozolomide-based regimens or cyclophosphamide–vincristine–dacarbazine) remains an option for rapidly progressive or high-burden disease, especially in metastatic settings (9). Therapy selection is guided by functional imaging phenotype (SSTR vs MIBG avidity), genetic background, tumor burden, progression rate, and symptom severity

In this patient, lanreotide therapy was associated with a marked decline in chromogranin A, stabilization of blood pressure, improvement in BNP, modest recovery of left ventricular ejection fraction, and reduced somatostatin receptor PET avidity, despite stable tumor size. These findings suggest that systemic control of neuroendocrine activity may translate into meaningful cardiovascular stabilization. While the improvement in ejection fraction was limited, even partial recovery may be clinically significant in advanced heart failure, particularly when accompanied by improved hemodynamics that permit optimization of guideline-directed medical therapy.

Although causality cannot be definitively established from a single case, the temporal association between lanreotide initiation and improvement across biochemical, imaging, and cardiac parameters is compelling and consistent with prior reports of reversible cardiac injury following reduction of catecholamine burden (2, 6, 12, 14). Importantly, the patient maintained excellent functional status without further heart failure hospitalizations, emphasizing the potential quality-of-life benefits of non-surgical systemic therapy.

In conclusion, this case supports a potential role for lanreotide as a non-surgical therapeutic strategy for PPGL-associated cardiomyopathy in select patients who are not candidates for tumor resection or transplantation. Multidisciplinary evaluation is essential, particularly in hereditary SDHB-associated disease. Further prospective studies are needed to clarify the cardiovascular benefits of somatostatin analog therapy and to define its role within the evolving treatment paradigm for PPGL-related cardiac disease.

## 4 Patient perspective

The patient expressed that his health had been declining for several years, particularly due to worsening fatigue and repeated hospitalizations for heart failure, which significantly limited his previously active lifestyle. He described frustration with the uncertainty surrounding his condition prior to diagnosis. After the discovery of the paraganglioma during transplant evaluation, he felt both surprised and concerned about the implications for his overall prognosis and treatment options. He carefully considered surgical intervention but ultimately chose to pursue medical management due to perceived risks and personal preference. Since starting lanreotide therapy, he reports improved stability in his symptoms, fewer limitations in daily activities, and no further hospitalizations, which he views as a meaningful improvement in his quality of life. He remains engaged in his care and expresses satisfaction with the current treatment approach

## Data Availability

The original contributions presented in the study are included in the article/Supplementary Material, further inquiries can be directed to the corresponding author/s.

## References

[1] Ikram A Rehman A Paraganglioma. In: StatPearls. Treasure Island (FL): StatPearls Publishing (2024). Bookshelf ID: NBK549834.

[2] Zhang J Cao L Yan L Jin C Zhang D. A young patient with heart failure was diagnosed with extra-adrenal paraganglioma: a case report. BMC Cardiovasc Disord. (2022) 22:574. 10.1186/s12872-022-03026-5 36581844 PMC9801580

[3] El Abidi H Ibrahimi A Mikou MA Iraqui I Boualaoui I Labbi Z. Heart failure induced by a tumor in a young adult woman: a case report of dilated cardiomyopathy triggered by paraganglioma. Radiol Case Rep. (2025) 20:3309–13. 10.1016/j.radcr.2025.03.024 40292142 PMC12018990

[4] Timmers HJLM Pacak K Huynh TT Abu-Asab M Tsokos M Merino MJ. Biochemically silent abdominal paragangliomas in patients with mutations in the succinate dehydrogenase subunit B gene. J Clin Endocrinol Metab. (2008) 93:4826–32. 10.1210/jc.2008-1093 18840642 PMC2626451

[5] Szatko A Glinicki P Gietka-Czernel M. Pheochromocytoma/paraganglioma-associated cardiomyopathy. Front Endocrinol (Lausanne). (2025) 16:123456. 10.3389/fendo.2023.1204851 PMC10374018 37522121

[6] Zhou J Xuan H Miao Y Hu J Dai Y. Acute cardiac complications and subclinical myocardial injuries associated with pheochromocytoma and paraganglioma. BMC Cardiovasc Disord. (2021) 21:203. 10.1186/s12872-021-02013-6 33882857 PMC8060996

[7] Y-Hassan S Falhammar H. Cardiovascular manifestations and complications of pheochromocytomas and paragangliomas. J Clin Med. (2020) 9:2435. 10.3390/jcm9082435 32751501 PMC7465968

[8] Yu Y Chen C Meng L Han W Zhang Y Zhang Z. Hypertension and cardiac damage in pheochromocytoma and paraganglioma patients: a large-scale single-center cohort study. BMC Cardiovasc Disord. (2024) 24:325. 10.1186/s12872-024-03936-6 38926862 PMC11200840

[9] National Comprehensive Cancer Network. NCCN Clinical Practice Guidelines in Oncology: Neuroendocrine and Adrenal Tumors. Version 1.2024. Fort Washington, PA: National Comprehensive Cancer Network (2024). 10.6004/jnccn.2021.0032 34340212

[10] Laderian B Zhou M Luk L Bates SE Fojo AT Del Rivero J. A phase 2 study of lanreotide as a therapy for pheochromocytomas (PCs) and paragangliomas (PGs). J Clin Oncol. (2025) 43(16_suppl):10612. 10.1200/JCO.2025.43.16_suppl.10612

[11] Kolasińska-Ćwikła AD Peczkowska M Michałowska I Pałucki J Roszkowska-Purska K Cichocki A. Biochemical and radiological efficacy of systemic lanreotide therapy of patients with advanced, unresectable, non-metastatic paraganglioma/pheochromocytoma (PPGL), sporadic and hereditary. Ann Oncol. (2024) 35(Suppl 2):S754–5. 10.1016/j.annonc.2024.08.1210

[12] Taïeb D Nölting S Perrier ND Fassnacht M Carrasquillo JA Grossman AB. Management of phaeochromocytoma and paraganglioma in patients with germline SDHB pathogenic variants: an international expert consensus statement. Nat Rev Endocrinol. (2024) 20:168–84. 10.1038/s41574-023-00926-0 38097671

[13] Lin FI Del Rivero J Carrasquillo JA Jha A Zou J Shamis I. Phase II study of 177Lu-DOTATATE for progressive metastatic pheochromocytomas and paragangliomas: interim analysis of efficacy, safety, and biomarkers. J Clin Oncol. (2025) 43:3102–12. 10.1200/JCO-25-00791 40829092 PMC12367064

[14] Giavarini A Chedid A Bobrie G Plouin PF Hagège A Amar L. Acute catecholamine cardiomyopathy in patients with phaeochromocytoma or functional paraganglioma. Heart. (2013) 99:702–7. 10.1136/heartjnl-2013-304073 23837998

